# Propofol-induced transient arginine vasopressin deficiency

**DOI:** 10.1530/EDM-24-0083

**Published:** 2024-10-28

**Authors:** Michael D Luppino, Huyen Nguyen, Matilda Smale, Rebecca Madigan, Morton G Burt, Mahesh M Umapathysivam

**Affiliations:** 1 Flinders University, Bedford Park, South Australia, Australia; 2 Southern Adelaide Diabetes and Endocrine Service, Flinders Medical Centre, Bedford Park, South Australia, Australia; 3 Department of Anaesthetic, Flinders Medical Centre, Bedford Park, South Australia, Australia; 4 University of Adelaide, Adelaide, South Australia, Australia

**Keywords:** Propofol, induced, transient, arginine vasopressin, deficiency

## Abstract

**Summary:**

We describe and characterise the case of a 26-year-old female undergoing surgery for a right-sided sinonasal alveolar rhabdomyosarcoma who developed profound, transient arginine vasopressin deficiency (AVP-D, formerly central diabetes insipidus (DI)) associated with anaesthesia. In this case report, we characterise the development of AVP-D with serial copeptin and paired urine and serum osmolality measurements. Based on the anaesthetic agent’s profile and the literature, we attribute this presentation to propofol exposure. We present a description of the literature on anaesthesia-associated DI as well as poignant learning points.

**Learning points:**

- Exposure to anaesthetic agents is a rare cause of self-limited but sudden and profound arginine vasopressin deficiency (AVP-D) or arginine vasopressin resistance (AVP-R).
- Sevoflurane has been associated with AVP-R and propofol with AVP-D, although the responsible agent may be difficult to identify.
- Differentiation of AVP-R and AVP-D can be made based on copeptin concentration, where available, or clinical response to desmopressin.
- Whilst the patient is anaesthetised, intravenous fluid replacement should be targeted to match urine output until the patient is able to drink to thirst. This should be clearly communicated to staff and the patient.
- Rapid resolution of AVP-R/AVP-D when the causative agent is discontinued has been reported with both propofol and sevoflurane. As such, switching the agent used to maintain anaesthesia may terminate increased urine output in a clinically meaningful timeframe.

## Background

This case describes a rare presentation of anaesthetic-induced arginine vasopressin deficiency (AVP-D) (formerly central diabetes insipidus (DI)). The presence of pre-operative incidental pituitary imaging, intraprocedural serial serum co-peptin, urine output, plasma and urine osmolarity allow new insights into the disease. The rotation of aesthetic agents which impacted urine output, also assists with certainty as to causal agents and potential efficacy of rotation of anaesthesia as a treatment strategy. We also report the response to subcutaneous desmopressin.

## Case presentation

**Figure 1:**
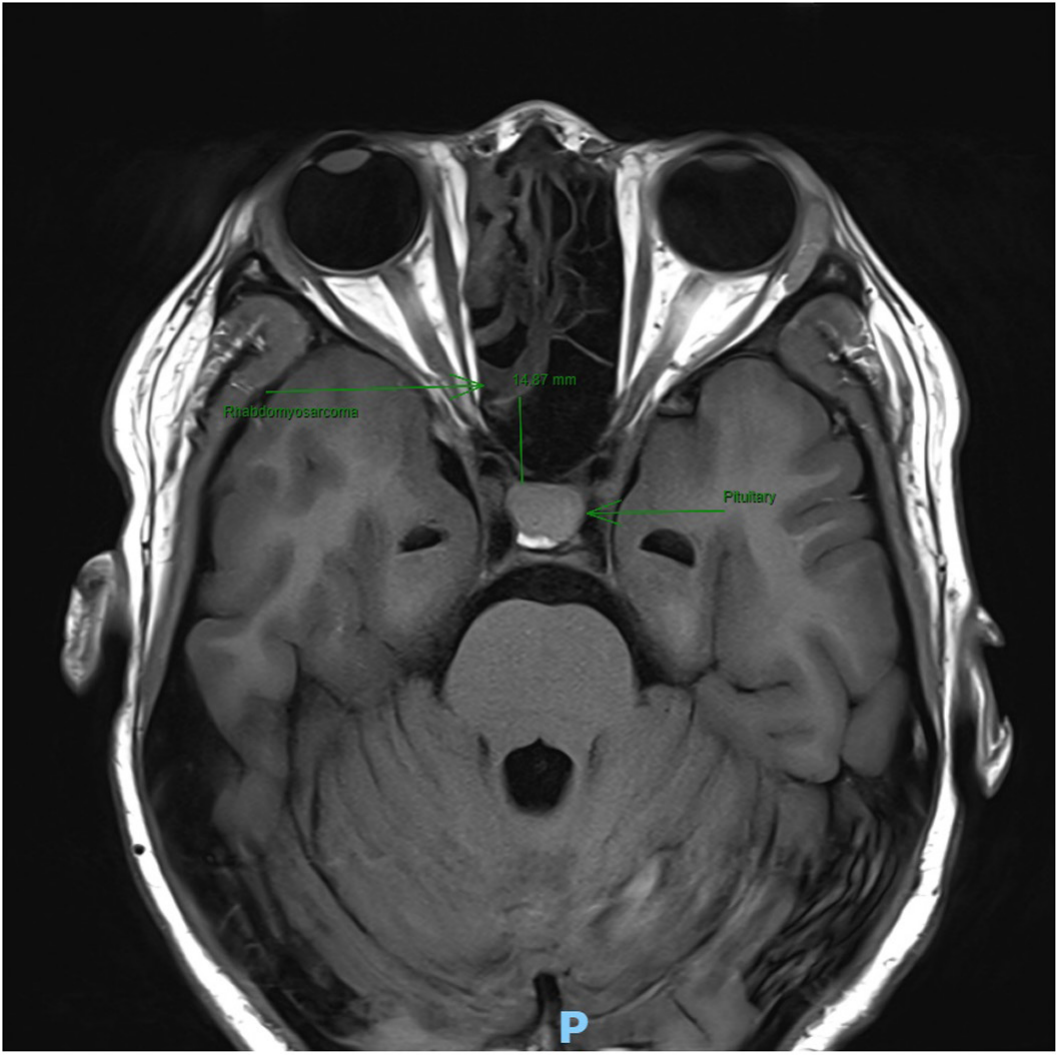
Pre-operative magnetic resonance imaging of the pituitary. The axial T1 weighted magnetic resonance imaging of skull base demonstrates the tumour is radiologically distinct and greater than 10 mm from the pituitary gland. The pituitary bright spot is intact.

A 26-year-old female underwent endoscopic resection of a right-sided sinonasal alveolar rhabdomyosarcoma (T2bN1M0) with extension into the orbital and parameningeal space. Prior to surgery, she had completed 10 cycles of neoadjuvant chemotherapy, which included vincristine, dactinomycin (actinomycin-D) and cyclophosphamide concluding 2 weeks before the procedure. Her pre-operative fluid intake was estimated to be 2–3 L daily. There was no prior anaesthetic exposure, and she had no notable family history of reactions to general anaesthetic. She had no other medical co-morbidities. MRI imaging prior to surgery demonstrated an intact pituitary bright spot on T1 imaging, consistent with a pre-operatively intact hypothalamic-neurohypophysis system (1). On MRI imaging, the tumour was >10 mm away from the pituitary gland (Fig. 1).

Anaesthesia was initiated with 200 mg of propofol, remifentanil 0.2 µg/kg/min and rocuronium 50 mg. She also received 8 mg of dexamethasone at induction. The nose was topicalised with cocaine and adrenaline (solution comprising 2 mL 10% cocaine, adrenaline 1 mL 1:1000 and 8 mL saline) and 2.2 mL lignospan special after intubation. Maintenance of anaesthesia was achieved with combined propofol target-controlled infusion (Marsh model) at 1.5–2 µg/mL and sevoflurane at an expired volume of 0.7–1.4%. A lumbar drain was then placed prior to the commencement of resection. The surgical procedure lasted 9 h.

**Figure 2:**
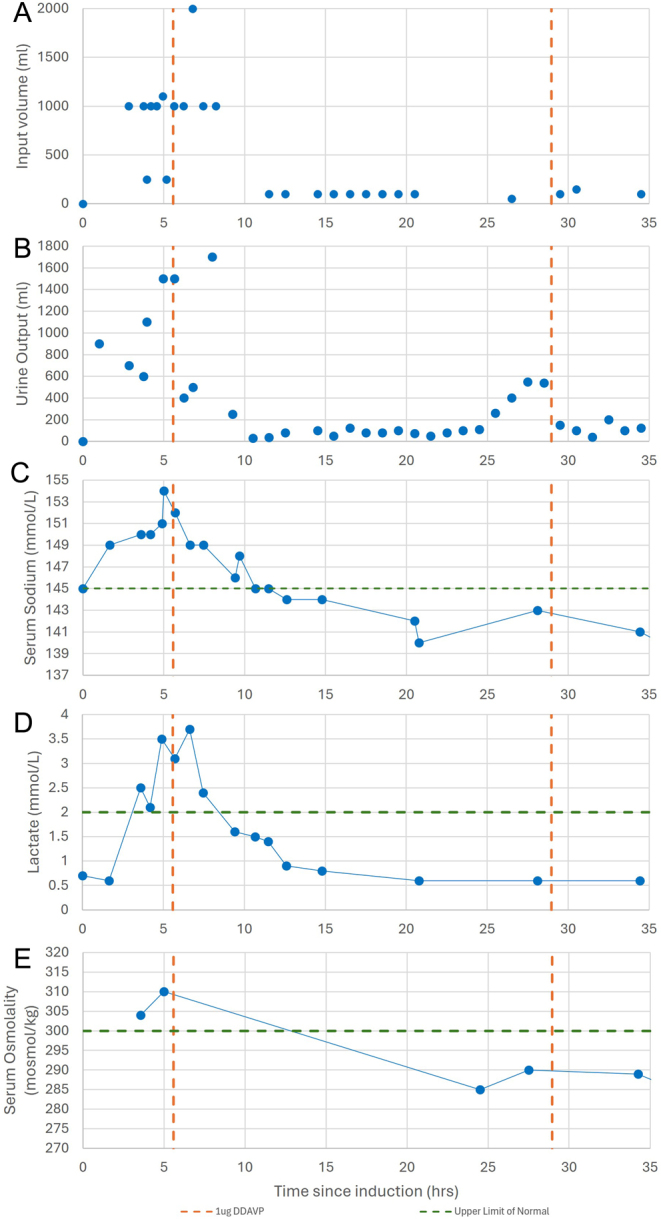
(A) Input volume represented as points signifying each bolus given at a given time, (B) output volume represented as a point signifying each time the catheter bag was emptied at a given time, (C) serum sodium, (D) serum lactate and (E) serum osmolality over time from induction (0 h) to 35 h. Note: the procedure duration was 9 h.

Shortly after induction but before surgical manipulation of the tumour, and prior to fluid administration, the patient developed increased urine output exceeding 550 mL/h (Fig. 2). This was accompanied by rising serum sodium (peak at 154 mmol/L), serum osmolarity (peak at 310 mosmol/kg) and serum lactate (peak at 3.7 mmol/L) (Fig. 2); the urine was dilute at 160 mosmol/kg.

From this, the diagnosis of arginine vasopressin (AVP) disorder was made. Over the course of her procedure, she excreted a total of 9150 mL of urine while receiving 11 602 mL of fluids, comprising two units of packed red blood cells (252 mL + 250 mL) and the remaining 11 100 mL as Hartmann’s solution (Fig. 2).

**Figure 3:**
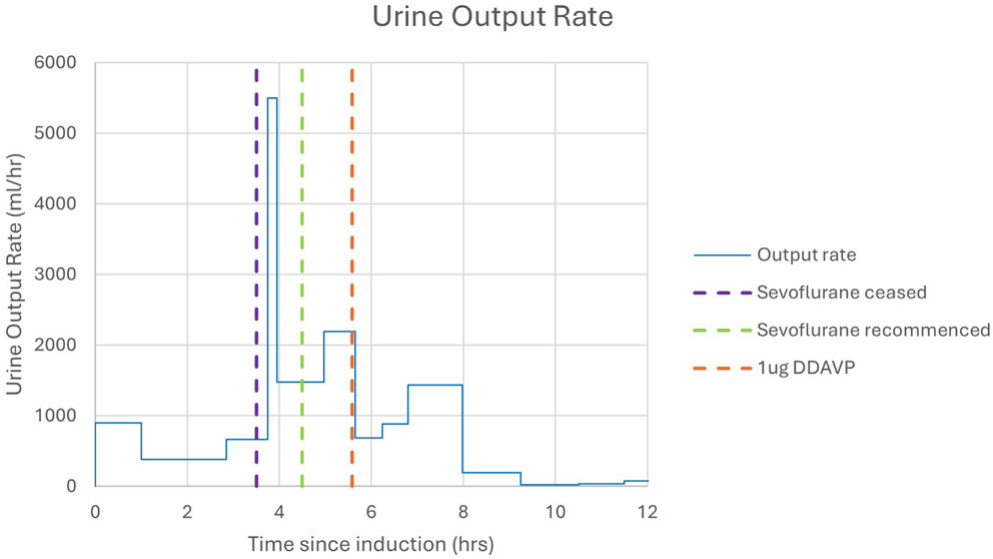
Relationship of anaesthetic agent to rate of urine output.

Three and a half hours after induction (during the surgery), sevoflurane administration was transiently interrupted due to concern it was causing AVP-D/AVP-R. During this time, the propofol infusion was increased to a plasma target of 4 µg/mL and the rate of urine output in this period further increased (Fig. 3), so sevoflurane was recommenced.

The copeptin level 5 h after induction was 0.8 pmol/L; this was in the presence of a serum osmolality of 310 mosmol/kg and serum sodium of 154 mmol/L, an osmotic stimulus sufficient to maximally stimulate AVP secretion. These findings are consistent with AVP-D. A baseline copeptin concentration <2.6 pmol/L with prior fluid deprivation (>8 h) indicates complete central DI is likely (2).

A partial pituitary panel was collected 5 h after induction (14:30 h). ACTH (<3 ng/L) and cortisol (30 nmol/L) were appropriately suppressed after 8 mg dexamethasone at induction. The thyroid-stimulating hormone was 1.41 mIU/L (0.5–4.5) and free T4 was 15 pmol/L (10–20). The repeat cortisol level on post-operation day 2 was 276 nmol/L at 10:15 h.

Five hours after induction, the patient was administered 1.0 µg of subcutaneous desmopressin, resulting in a reduction but incomplete normalisation of urine output; the urine output slowed from 1500 mL in the hour prior to desmopressin, to 500 mL within an hour (Fig. 2) Urine output remained between elevated until 2.5 h after administration of desmopressin (hour 8 of procedure) when it fell to <100 mL/h.

Following surgery, the patient’s urine output was monitored hourly in intensive care. Twenty-five hours after induction, at least partial recovery of AVP secretion was suggested by copeptin rising to 4.1 pmol/L with a serum osmolality of 285 mosmol/kg and serum sodium between 140 and 143 mmol/L. At 29 h post induction, urine output increased to 540 mL/h with an associated urine osmolality of 126 mosmol/kg; she was administered a second dose of 1.0 µg of desmopressin subcut. There was again a response to desmopressin, consistent with a central rather than nephrogenic pathology.

The patient was instructed to drink to thirst and provided free access to water. Her urine output was monitored for the following 72 h with no further evidence of increased urine output. Serum osmolality normalised on day 1 post-operatively. Following resolution, the copeptin ranged from 0.8 to 4.1 pmol/L but was sufficient to maintain urine output under 3L daily.

## Discussion

Anaesthesia-induced AVP disorder is a potentially life-threatening syndrome as patients are vulnerable to the effects of urinary fluid loss and volume depletion without the protective mechanism of thirst. We present the case of a 26-year-old female with anaesthesia-associated transient AVP deficiency. The diuresis was profound and if untreated would have resulted in haemodynamic compromise. Even with aggressive fluid replacement, there was evidence of under perfusion with a rising lactate.

The partial response to desmopressin in our case made it initially ambiguous to determine if the cause was AVP-R or AVP-D; a copeptin was needed to differentiate between these two causes. Copeptin is derived from the C-terminal segment of AVP and is secreted in a 1:1 ratio with AVP. It is diagnostically more reliable than measuring AVP due to its *ex vivo* stability (2). In our case, a low copeptin at a time of maximal osmotic stimulation of AVP secretion and partial response to desmopressin confirmed AVP-D rather than AVP resistance (AVP-R).

Anaesthesia was maintained with both propofol and sevoflurane for the duration of the surgery, with the caveat of the sevoflurane cessation 3.5 h into the 9-h surgery, with subsequent recommencement 1 h later. During the period of sevoflurane cessation, the propofol infusion rate was increased with a corresponding increase in urine output, suggesting propofol, not sevoflurane, was the causative agent (Fig. 3).

Within the literature, there are two reported cases of propofol-induced AVP-D. Kassebaum *et al.* reported a case in which desmopressin administration normalised post-operative urine output, plasma osmolality and plasma sodium (3). The patient had follow-up testing that demonstrated normal osmotic regulation. Desmopressin responsivity in this case supports the observations that propofol-induced AVP dysfunction is driven by AVP-D and was seen to be transient.

Further support for the transient nature of propofol-related AVP-D is offered by Soo *et al.*, who present a patient who was exposed to both sevoflurane and propofol (4). In this case report, switching the anaesthetic agent to propofol during the case was associated with a dramatic increase in urine output. Cessation of the propofol infusion and switching back to sevoflurane resulted in a prompt reduction in urine output. This is similar to the pattern observed in our case. The patient in the case reported by Soo *et al.* was not administered desmopressin nor were copeptin levels measured.

Clues to a potential mechanism of action for propofol-induced AVP-D are offered by previous murine models (5). Inoue *et al.* demonstrated that propofol inhibited the secretory capacity of cells of the supraoptic nuclei (which produce AVP) when stimulated by either potassium chloride or glutamate. However, to date, similar studies have not been replicated in humans.

Case reports of sevoflurane-induced AVP dysfunction have not responded to desmopressin, and some even had persistent symptoms for days after the procedure (6, 7). This suggests a likely nephrogenic cause (AVP-R). This deduction is supported by a study performed by Morita *et al*, which demonstrated that sevoflurane temporarily suppressed urinary expression of AQP2 and reduced urine concentrating capacity (8).

A case presented by Schirle reported an almost immediate normalisation of the AVP-R when sevoflurane was ceased. Here, the duration of sevoflurane was less than 2 h, far shorter than previously described sevoflurane cases (6, 7, 9). Together, these cases may indicate a duration-dependant relationship between sevoflurane exposure and recovery time of the polyuria.

At the cessation of anaesthesia, our patient had at least partial recovery of AVP secretion, however, she did require a second dose of desmopressin 29 h after induction, suggesting an ongoing effect (Fig. 2). In keeping with the available literature, the duration of polyuria cannot be used to accurately determine the causative agent but may correlate with the duration of anaesthetic exposure. Prior cases of both propofol and sevoflurane-induced AVP dysfunction have demonstrated rapid reversal of symptoms on cessation of the anaesthetic agent when there was a short drug exposure (4, 9). Given the reported rapid response to discontinuation of causative anaesthetic agents, it may be a viable management option to switch or rotate the agent used to maintain anaesthesia and wait for a reduction in urine output, although this is likely to only be of use in protracted procedures.

Dexamethasone was the other medication administered that potentially can affect AVP. Glucocorticoids inhibit AVP secretion and can unmask AVP deficiency in a patient with concomitant anterior and posterior pituitary dysfunction (10). However, this patient was unlikely to have glucocorticoid deficiency as morning cortisol was normal 2 days after dexamethasone, the mass and surgery were separate from the pituitary gland, and there was no evidence of dysfunction of other pituitary axes. Rocuronium and remifentanil were administered at induction, but there are no reported cases of AVP dysfunction associated with their prescription. While we cannot be certain of any contribution, this is less likely.

This rare case of propofol-induced AVP-D is supported by a transient reduction in AVP secretion evidenced by low intra-operative copeptin levels and normalisation of urine output, urine osmolality, plasma osmolality and plasma sodium levels with desmopressin. The patient was successfully supported with subcutaneous desmopressin and intravenous and oral fluids until the return of her capacity to secrete AVP and concentrate her urine.

## Declaration of interest

MM Umapathysivam received grants from Diabetes South Australia (SA) and grants from Australian Diabetes Society funded by AstraZeneca outside the submitted work. The other authors have no conflicts of interest to declare.

## Funding

This work did not receive any specific grant from any funding agency in the public, commercial or not-for-profit sector.

## Patient consent

Written informed consent for publication of their clinical details was obtained from the patient.

## Author contribution statement

MDL drafted the manuscript and extracted relevant data from the medical record; HN and MS assisted with co-ordination of sample collection and reviewed manuscript; RM identified the case and reviewed the manuscript; MGB assisted with interpretation of investigations and reviewed manuscript; MMU conceived the manuscript and assisted with manuscript drafting and review and data interpretation and was the physician responsible for the patient. MMU had full access to all of the data in the case report and takes responsibility for the integrity of the data and the accuracy of the data analysis.
